# Development of a Pandemic Residual Risk Assessment Tool for Building Organizational Resilience within Polish Enterprises

**DOI:** 10.3390/ijerph18136948

**Published:** 2021-06-29

**Authors:** Tomasz Ewertowski, Marcin Butlewski

**Affiliations:** Faculty of Engineering Management, Poznan University of Technology, 2 Prof. Rychlewskiego Str., 60-965 Poznan, Poland; tomasz.ewertowski@put.poznan.pl

**Keywords:** risk assessment, occupational health and safety, organizational resilience, biological hazard

## Abstract

The purpose of the research paper was to develop a universal residual risk assessment tool based on the use of risk control measures related to Covid-19 in order to determine the state of organizational resilience of individual industries or organizations. The article proposes and analyzes a pandemic residual risk assessment tool, which is a simple and universal source for residual risk estimation based on a five-step consequence/probability matrix, a five-step hierarchy of risk controls, and a general formula for calculating residual risk. The methodology of the survey is based on a questionnaire with 16 questions used for the initial validation of the residual risk scale, of which six related to the potential of organizational resilience. The pilot survey was conducted in 66 enterprises in Poland. On the basis of the survey, four measures related to the use of control measures against threats after the outbreak of the Covid-19 pandemic have been proposed. These are personal protective equipment (PPE) controls, administrative controls, engineering controls, and substitution controls. Using the survey results, we estimated averages of the response results, and, on their basis, we estimated the residual risks for individual types of enterprises according to the type of business and its size. Based on the calculations, a strong correlation was found between the potential of organizational resilience and the individual use of control measures. Therefore, the main finding of the survey proves that effective risk management builds organizational resilience in enterprises. The practical implications of the study allow the management staff to find out what aspects related to the use of control measures need to be paid attention to in order to minimize residual risk.

## 1. Introduction

The outbreak of the Covid-19 pandemic undoubtedly caused a crisis in the economies around the world. We define a crisis as an extreme destabilization of the functions of an organization. On average, it can be assumed that the most severe phase of the crisis lasts several months depending on the situation and organization. A crisis situation, on the other hand, takes much longer and may last up to several years. A crisis situation is also a multi-phased period in which at least three phases can be distinguished as different from a period of normal operation: pre-crisis (escalation), crisis, and post-crisis (recovery) [1]. Aside from the causes of crises, which might be terrorism, social and financial crises, pandemics, or natural disasters [2], every crisis has the power to damage businesses, which have to adapt to the new environment and adjust to different situations every time [3]. Crisis management focuses on the financial stability of a business and on the protection of the working environment and employees. Unprecedented global transport restrictions and stay-at-home orders connected with the pandemic have caused the most severe disruption of the global economy since World War II [4]. The pandemic had repercussions for the entire economy and at the same time revealed a different level of organizational resilience of enterprises, depending on their sector [5] or size [6]. The global crisis revealed the need for macro-economic and multisectoral collaboration to survive during the most severe phases of the crisis and the times when the most stringent global protective measures are taken [7]. The assessment of the ability of enterprises to function in individual phases of the global crisis allows for the development of tools and scales of organizational resilience. Brown defines organizational resilience as “the ability to survive a crisis and thrive in a world of uncertainty” [8].

The aim of the study was to develop a universal residual risk assessment tool based on the use of risk control measures related to Covid-19 in order to determine the state of organizational resilience potential of individual industries or organizations as well as to refer to collective risk measures. In line with the idea of residual risk, enterprises should balance safety measures with the boundaries adopted by them. The balance between the remaining risk and the measures used is crucial for the efficient operation of the organization in the face of global threats.

## 2. Literature Review

Organizational resilience considers physical properties as well as organizational structure and capacities [9,10]. Brown and colleagues [8] state that a resilient organization should detect unexpected events early and then must develop resilience capabilities to react to the negative consequences of unexpected events and to return quickly to its original state, the one before risk occurrence, or to move to a new best state after being affected by the risk and continue business operations as efficiently as possible. The root word of resilience comes from the Latin ‘to bounce back’. Another of the more widespread in the economy is the definition included in the standard ISO 22316: 2017 [11], which describes organizational resilience as the “ability of an organization to absorb and adapt in a changing environment”. The adaptive resilience approach mitigates harm or utilizes opportunities based on redevelopment. This adaptability makes an organization stronger against crises and works like a vaccine. Adaptation can be grouped into four R categories of possible actions: Redundancy, Resourcefulness, Robustness, Rapidity. Redundancy is the ability of an organization to provide uninterrupted services in the event of a disruption; Resourcefulness is the utilization of materials (human resources, financial, technological and informational) to establish, prioritize, and achieve operational goals. Robustness is the strength or the ability of the system to resist disturbance-induced stressors. The Rapidity is the capacity to return the system to a predisturbance level of functioning as quickly as possible [12]. Shilari with colleagues [13] states that the concept of resilience has been applied in several fields, such as ecology, social and organizational science, psychology, computer science, etc. It is important that the meaning of resilience generally remains similar across applications.

Organizational resilience is described through four key organizational skills [14]:

a) responding, defined as skills and knowledge of what to do and how to respond to regular or irregular disturbances and crises by either introducing previously prepared actions or by adapting normal functioning to a changing situation,
b) monitoring, defined as skills and knowledge of what to look for, what could be or become a threat in the near future; monitoring must include both the organization’s environment and its own activities,
c) anticipating, defined as skills and knowledge of what to expect in terms of development, threats and opportunities in the future, e.g., potential changes, disruptions, pressure and consequences of the mentioned factors,
d) learning, defined as skills and knowledge derived from both positive and negative experiences.

Organizations need to integrate elements of resilience into their everyday business practices to improve their response in the face of adversity [15].

Risk awareness and its proper management is the best way to prevent and slow down the transmission of the Covid-19 pandemic and build organizational resilience. The management and other employees should be aware of the limits of acceptable and tolerable risk, and immediately take preventive measures when exceeded. There are many definitions of risk. One of the most widespread in the economy is the definition contained in the standard ISO 31000: 2018 [16], which describes risk as “the impact of uncertainty on objectives”. On the other hand, risk management is defined as “coordinated activities aimed at directing and controlling the organization in relation to risk”. The International Organization for Standardization (ISO) defines this standard as a framework for managing various risks in various sectors of the economy. However, each of these sectors should adapt the relevant concepts to manage the specific risk involved. Complementing the ISO 31000 in the issues raised is the ISO IEC 31010: 2019 Risk Management—Risk Assessment Techniques standard [17]. This standard provides a set of guidelines for the selection and application of risk assessment techniques in a wide range of situations. These techniques are used as a decision aid in case of uncertainty, in providing information on individual risks and as part of the risk management process. Shaw with colleagues [18] argue that the pandemic is global, but that its response is local. They also indicate that a significant proportion of the responses depend on the organization, its culture and the behavior of employees. In the literature, we can find many examples of risk assessment dedicated to Covid-19, focused on new outbreaks [19,20], and the efficiency of different safety means [21]. No dimension of the impact of the Covid-19 epidemic on socio-economic life was possible without a series of articles commenting on it. Some of them concern the methods of individual risk estimation through the use of web applications [22]. Although they raised doubts about social acceptance at least in part [23], in the present situation it is much easier to articulate the need to ensure the safety of the general public, which limits individual freedom. These activities include, for example:

- tracking people who are infected or potentially likely to be carriers,
- provision of services depends on the result of the risk analysis for individual people (e.g., refusal in the case of signs of disease),
- tracking goods and human flows in order to analyze global risk and assess the effectiveness of security measures taken by both state and private operators.

The situation may also affect a number of everyday behaviors, such as exchanging a handshake or paying in cash, but the durability of these changes will depend on the aggregate effectiveness of anti-virus activities. Ranit with colleagues [24] presented the risk assessment method “The Covid-19 Risk Assessment Tool”. It is a simple online tool that allows you to assess your risk based on many interrelated factors. It is essentially based on four main factors: health status, exposure, behavior represented, and social policy. Each of these agents contains ingredients that are based on the results of studies conducted in China and Italy [25]. Another example of this type of application is the Henry Ford Health System [26], where the app asks questions about symptoms, travel history, and contact for risk analysis. Other methods of Covid-19 dedicated risk analysis are based on estimating the effectiveness of control measures such as personal protective equipment (PPE) or technical or administrative methods. Nur et al. [27] argue that personal protective equipment should be used as primary prevention, which, unfortunately, in the absence of technical and administrative methods of risk control, constitutes limited protection options. Even in specific areas of entrepreneurship such as the hospitality industry, which, as one of the most affected industries by the epidemic, mention practices that are generally well-known methods of counteracting the spread of the epidemic, extended to those available at the level of state administration [28,29]. Ali with colleagues [30] stated that, the main factors in the event of infection include a lack of understanding of the disease, inadequate use and availability of Personal Protective Equipment (PPE), uncertain diagnostic criteria, unavailability of diagnostic tests, and psychological stress.

## 3. Tools and Methods

The residual risk is the risk that remains after all possible or partial control measures and best practices for dealing with it have been applied. In the context of the pandemic, the best practices were associated with efficient utilization of the hierarchy of controls triangle [31], detailed by the authors in the survey questions, based on an analysis of the recommendations of the State Sanitary Inspection and an analysis of the recommendations of the World Health Organization (WHO) regarding the implementation of the so-called sanitary regime [32]. The main practices to combat COVID-19 included:

- Provide water and soap or 70% alcohol; self-reported symptoms form; personal protective equipment in terms of surgical face masks,
- Organize the workplace to maintain a safe distance (1.5 m) between workers, considering the guidelines of the Ministry of Health and the characteristics of the work environment,
- Prioritize measures to distribute the workforce throughout the day, avoiding concentrating it in just one shift,
- Disinfect workplaces and common areas between shifts or whenever a worker is designated to occupy another person’s job,
- Provide emergency care to suspected or confirmed cases of COVID-19 [33].

**Figure 1:**
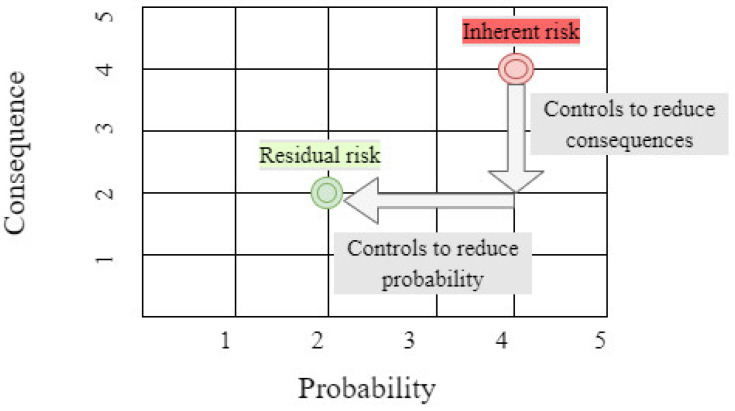
The risk reduction process.

It helped the authors to design the questionnaire. Even if all theoretically possible safety measures are implemented, this is the risk that remains after the management of the company or another organization takes actions to minimize the impact (effects) and the probability of adverse events, including control actions taken in response to the risk [34]. In order to reduce the level of risk, the probability of risk occurrence and its consequences should be reduced or eliminated. This is done with the most effective control measures. This in turn will reduce the risk to an acceptably low level with as little residual risk as possible. This mechanism is presented in Figure 1.

The general formula for calculating the residual risk is

(1) Residual  Risk = Inherent Risk −Impact of Risk Controls 

where the overall concept of risk is consequence × probability [34]. When risk is considered at the design or development stage, risk elimination and replacement can be inexpensive and easy to implement. An existing process may require major changes to equipment and procedures to eliminate or replace a hazard. In the case of Covid-19, the first of the most effective areas of impact on a biological threat, which is elimination, is currently not possible (the introduction of vaccination gives some hope related to this). Therefore, solutions from the remaining control measures should be sought even more. Technical controls are preferable to administrative and personal protective equipment (PPE) for controlling existing worker exposure in the workplace as they aim to remove the hazard at the source before it comes into contact with the worker. Well-designed engineering controls can be very effective in protecting workers and will typically be independent of worker interactions to ensure this high level of protection [35]. The initial cost of engineering controls may be higher than the cost of administrative controls or PPE, but in the long run, operating costs are often lower. Administrative control measures and PPE are often used in existing processes, where risks are not particularly well controlled. Administrative controls and PPE programs can be relatively inexpensive to set up, but in the long run they can be very costly to maintain. These methods of protecting workers have also proved less effective than other measures, requiring considerable effort on the part of the workers concerned. The method of using PPE is, however, influenced by a number of different factors, such as a safety culture, the measurement of which requires many different research procedures [36].

The developed method of residual risk assessment is based on a 5-step consequence/probability matrix, a 5-step hierarchy of risk control measures and a general formula for calculating residual risk. To determine the probability, a qualitative (descriptive) scale was adopted as shown in Table 1. The probability scale was adjusted to the observed frequency of Covid-19 cases in Polish organizations during the second wave of Covid-19 in the fall of 2020.

The adopted qualitative scale of probability corresponds to the number of people infected with Covid-19, provided by companies during audits and interviews, as well as the data on the number of Covid-19 cases. The consequences scale was adopted and modified by the authors from the Government Security Center (RCB, 2019) [37]. To determine consequences, its severity, classification and characteristics were analyzed in three dimensions, i.e., H&L—health and life, F—finance in EURO, E&S—environment and society, presented in Table 2:

The risk control hierarchy scale is based on the commonly used triangle of risk control hierarchy [31], as shown in Table 3.

**Figure 2:**
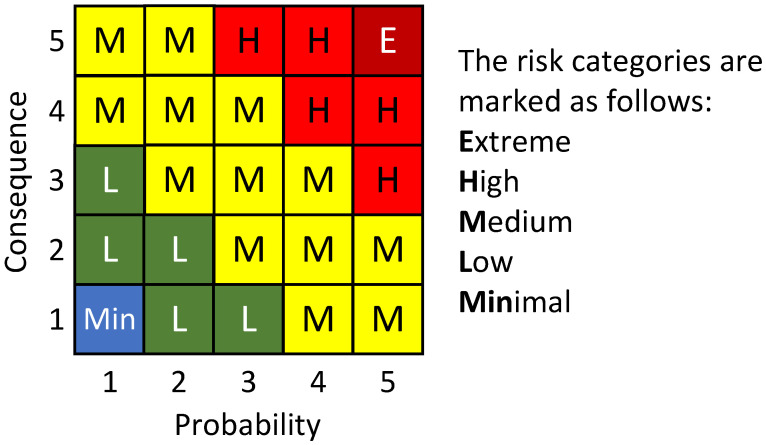
Risk matrix of the developed method.

After determining the probability and the impact, it is possible to indicate the value of the risk. The risk value for each scenario is indicated in the risk matrix showing the relationship between the probability and the consequences, as shown in Figure 2.

We calculate the risk level according to the formula (2)

(2) $$Risk\;\;Level=\sqrt{Probability*Consequences}$$

Next, the residual risk is calculated according to Formula (1). It can be assumed that the acceptable level of residual risk is ≤2. Measures of 5 categories, based on questions, each rated on seven-point Likert-type scales (from “strongly disagree” to “strongly agree”) allow us to obtain the resulting data for the estimation of arithmetic mean values of individual measures of the use of control measures.

Then, the arithmetic weighted average taking into account the results on the 7-point Likert scale for the 5-point scale depicted in Table 3 is expressed by the formula:

(3) $$\overset{-}{s}=\frac{5}{7}\left(\frac{w_{p}s_{p}+w_{a}s_{a}+w_{e}s_{e}+w_{s}s_{s}+w_{el}s_{el}}{w_{p}+w_{a}+w_{e}+w_{s}+w_{el}}\right)$$

where: *s_p_* = PPE average, *s_a_* = average of administrative means, *s_e_* = average of technical means, *s_s_* = average of substitution means and *s_el_* = average of elimination means, *w_p_*, *w_a_*, *w_e_*, *w_s_*, *w_el_* weighting factors, which were respectively taken as: *w_p_* = 1, *w_a_* = 2, *w_e_* = 3, *w_s_* = 4 and *w_el_* = 5.

After developing a tool for assessing the Covid-19 residual risk, we tried to initially validate the tool.

The first step in validating a survey was to establish face validity. The questions were assessed to the two university administration employees connected with the internships in order to evaluate whether the questions effectively capture the topic under investigation and, to check the survey for common errors like double-barreled, and leading questions [38].

The second step was to pilot test the questionnaire on a subset of the intended population. For this reason, an objective assessment of the application of individual measures is important, preferably carried out by an independent expert. To overcome this difficulty, taking into account the specific situation and time constraints, student internships were used, during which adepts of management, logistics and safety engineering of the Faculty of Engineering Management at the Poznan University of Technology had the opportunity to conduct audits in enterprises. Each student could start an internship in one of about a thousand companies registered for cooperation with the Poznan University of Technology. The justification for conducting the research by students undergoing internships in the surveyed enterprises results from at least several reasons. The argument for such a procedure was, first of all, unique access to the enterprise at a time when the enterprises limited any contacts or visits. Students were required to thoroughly analyze the company’s processes and submit a written report, which greatly facilitated answering questions about measures in the field of organizational resilience. The research was conducted after the sixth semester of studies, to check with “their eyes” the use of control measures related to the reduction of the Covid-19 pandemic risk and organizational resilience. They were well prepared by the researchers because they had known the risk assessment methods and control measures from lectures. In addition, the questionnaire was accompanied by a cover letter, explaining the purpose of the research. The questions were prepared so that they were clearly understood and each of them had an appropriate explanation. It was a fairly unique opportunity to gain insight into the activities of companies in the field of defense against the biological threat associated with Covid-19, not through questions directed to companies, but through internal observers. The data collection procedure was conducted in the period between 25 October 2020 and 5 December 2020. The study period in Poland coincided with the second wave of Covid-19 cases, which was much more severe. The questionnaire was sent to the 184 students of the Faculty of Engineering Management. The 66 completed questionnaires were received during the research–Table 4. After they filled out the form one tried to point out which questions were weak or irrelevant. No questions were dropped.

The third step of the initial validation was associated with a checking the internal consistency of questions loading onto the same scales. This step basically checks the correlation between questions loading onto the same factor. One used the Spearman correlation coefficients because it evaluates the monotonic relationship between two continuous or ordinal variables (the data set has not the normal distribution—The Shapiro–Wilk test was performed and *p* < α, the α- level of significance considered was 0.05). The item-total correlation varied from 0.21 to 0.73. It means that the strength of the correlation was from weak (0.2–0.39) to strong (0.6–0.79), but mainly values were in moderate or strong interval of strength [39]. One used the overall Cronbach’s alpha coefficient for a measure of reliability and the internal consistency of questions. The overall Cronbach’s alpha coefficient of controls measurement was 0.92. As α CR were higher than 0.70, it was confirmed that the questionnaire is reliable for data evaluation [40]. In this way, a high value of internal consistency was obtained.

Despite the initial validation carried out in this three steps way, the study has limitations described in the discussion paragraph.

The categorization of the size of an enterprise (micro, small, medium or large) is made on the basis of the number of employees and financial data (net income and balance sheet total). This categorization is universal for all of Europe and used according to Commission Recommendation of 6 May 2003 concerning the definition of micro, small and medium-sized enterprises (Journal of Laws UE L 124 of 20.05.2003, p. 36) [41]. Size categories are as follows: Micro (from 1 to 9 employees and annual turnover of less than 2 million EUR). Small (from 10 to 49 employees OR annual turnover of 2–10 million EUR), Medium (from 50 to 249 employees OR annual turnover of 10–50 million EUR), Large (more than 249 employees OR over 50 million EUR of annual turnover). Due to the necessity of reporting, each company knows the assigned category, therefore its determination does not pose any major problems. In further analysis, when comparing sectors outside industry, such as: transport, construction, trade, insurance services, etc., they were combined into one group, creating the category: other. This was necessary due to the small number of enterprises from particular sectors. The collected data was coded and checked for correctness and randomness of the answers provided. The STATISTICA 13 program was used in the data analysis. The main statistical methods used in this study included: descriptive statistics to compute summary statistics such as means or standard deviations, descriptive statistics by groups to calculate descriptive statistics and correlations for dependent variables in each of a number of groups defined by one or more grouping (independent) variables, reliability and item analysis to assess internal consistency of items and Cronbach’s alpha coefficient. The research utilized some qualitative and quantitative questions. In this paper, the results of semi-qualitative assessment would be considered using a questionnaire. The aim of the study was to measure the use of risk control measures related to Covid-19 in selected Polish enterprises and to measure their organizational resilience potential. The questionnaire consisted of 16 questions each rated on seven-point Likert-type scales (from “strongly disagree” to “strongly agree”). These questions were then selectively assigned to specific control measures: PPE, Organizational, Technical and Substitution, and to the category of organizational resilience potential. In the case of Covid-19, the first of the most effective measures to address biological hazards–elimination–is currently not possible, because it would have to involve the cessation of activity by the enterprises or the vaccination of employees (which was not yet available at the time of the conducted research). For the purposes of elimination, the response value was assumed at level 1.

## 4. Results

The following are the identified statements that describe companies’ strategies for using control measures against the threat of a pandemic. The overall Cronbach’s alpha coefficient of controls measurement was 0.92. However, the item-total correlation varied.

The obtained results were further elaborated in order to obtain a tool for calculating a residual risk assessment scale.

Based on the obtained results of weighted averages for individual grouping variables, after the traditional calculation of probabilities (Table 2) and consequences (Table 3), we obtain results that allow us to calculate the residual risk in accordance with Formula (1). The list of items presented in Table 5 was used to build the scales of the hierarchy of risk control measures and the potential of organizational resilience as well as the scales of individual dimensions of residual risk in enterprises. Table 6 provides a list of items in individual scales.

The period of the study and student internships in Poland coincided with the second wave of Covid-19 cases, which was much more severe than the first one, and there were from 382 to as many as 27,086 cases per day during this time., Therefore, for the purpose of estimating the residual risk according to the developed method, the following risk parameters were adopted: probability—very common, but the consequences—high. This resulted in an inherent risk index value of 20. For this assumed average level of inherent risk, the following residual risk results presented in Table 7 were calculated on the basis of the obtained research results using the formulas (1,2,3).

The results of the Spearman correlation between the total use of control measures and the organizational resilience of the examined enterprises are depicted in Table 8.

## 5. Discussion

In this study, a new pandemic residual risk assessment tool is proposed for qualifying the residual risk of an enterprise. The proposed tool is based on a five-step consequence/probability matrix, a 5-step hierarchy of risk controls, and a general formula for calculating residual risk. For the initial validation of the residual risk scale, a questionnaire with sixteen questions was used, 6 of which related to the potential of organizational resilience, each rated on seven-point Likert-type scales (from “disagree strongly” to “agree strongly”). On the basis of the survey, four measures related to the use of control measures against threats after the outbreak of the Covid-19 pandemic have been proposed. These include PPE controls, administrative controls, engineering controls, and substitution controls. Using the survey results, we estimated the average response results and on their basis, the residual risks for individual types of enterprises in terms of the type of business (industrial and other) and the size of employment (small, 1–49 employees; medium, 50–250 employees and large, over 250 employees). The best in terms of the use of control measures were enterprises related to other types of activity (construction, trade and services), whose average, calculated in accordance with the proposed formula, was 2.71 against 2.50 in industrial enterprises. The results were associated with the degree of implementation of the compulsory sanitary regime in enterprises. More restrictions were imposed in trade and service companies than in industrial ones because this branch had to serve more external customers. These two groups of companies were connected with close and frequent contact with visitors, suppliers, and co-workers.

The same criterion in terms of the use of control measures was also best used by large plants (2.66) before medium (2.63) and small plants (2.48). It was connected with better resources and a more systemic approach presented by larger plants. Of the control measures, administrative control measures (5.64) ranked before technical (5. 43) and PPE (4.82) and finally Substitution controls (4.42). The responses for Elimination controls were assumed to be 1 on the Likert scale due to the inability to eliminate the hazard during the study. Then, for the purpose of estimating the residual risk in accordance with the adopted method, the following parameters of inherent risk were averaged for a specific pandemic situation during the study in Poland: probability—very common, while its consequences are large. This finally gave the inherent risk index value of 20. For this assumed average level of inherent risk, on the basis of the obtained research results, the results of residual risk were calculated using appropriate formulas, which were best for enterprises related to other types of activity (1.76) compared to 1. 97 in industrial enterprises. The same criterion was also best used by large plants (1.81) before medium (1.84) and small plants (1.99). As can be seen, the residual risks were on the border of the proposed acceptability of the tool (residual risk ≤ 2). This proves that enterprises were in a very difficult operating situation and that even the slightest disturbance related to the pandemic could increase the risk to an unacceptable level. Due to the development of vaccines with 60–90% effectiveness, and their introduction to use after the study described in the article, they were not included in the survey. On the other hand, having this knowledge, at the time of preparing the article, vaccinations were treated as a control measure to eliminate the risk and in the calculations, hypothetical responses were simulated at the level of a mean Likert scale 3.0, 4.0, and 5.0 respectively. On this basis, the total use of control measures was obtained at the level of means calculated in accordance with the formula, i.e., 3.08, 3.17, and 3.55. This resulted in final results for the residual risk at the level of 1.39, 1.30, and 0.92, respectively. There is a significant improvement in reducing the residual risk to a widely acceptable level, which corresponds with ALARP (as low as reasonably practicable). This principle generally requires that the level of risk be kept reasonably low, but this may mean different levels in different industries or occupations.

The added value of the study was the inclusion of questions concerning the measurement of the organizational resilience potential in the surveyed enterprises. Based on the analysis of its results, it can be concluded that the highest potential for resilience was shown by enterprises related to other types of activity (5.35) compared to 5.07 in industrial enterprises. According to this criterion, the best enterprises were large (5.40) before medium-sized (5.21) and small (4.92). In addition, the Spearman’s rank-order correlation between the organizational resilience potential and the individual use of control measures was calculated. Based on the calculations, a strong correlation between the parameters was found, amounting to 0.75. It can be concluded that the better the use of the hierarchy of control measures in an enterprise, the greater the resilience potential. Therefore, effective risk management builds organizational resilience in enterprises. Thanks to such a developed tool, it is possible to conduct a multi-criteria analysis of the use of control measures and the related residual risk. The proposed tool was initially positively verified by a pilot survey of 66 companies in Poland. It is anticipated that this tool can be universally applicable to risk assessment of other threats and their impact on the resilience potential of an organization.

This research comes with some limitations. This study is not conclusive because of its sample size and findings can be incomplete and may not be generalized. Due to the changing dynamics of the current pandemic, the presented tool allows the management staff to find out what aspects related to the use of control measures need to be paid attention to in order to increase the resilience of the organization. The research took place during the second wave of Covid-19, which was much more severe than the first one. For this reason, it was not possible to use the tool in companies from some industries. Some industries like hospitality were completely shut down and were therefore unable to implement the Organizational Resilience Plan. As it was mentioned in “Tools and method” that each student could undergo an internship in one of about a thousand companies registered for cooperation with the Poznan University of Technology. This may be considered that there could have been a selection bias. However, we believe that the surveyed companies provide sufficient statistical representativeness for initial validation of the tool This makes the findings an important element of discussion in the development of resilience by companies in the future. However, because of these limitations up to now, the tool has not been fully validated and additional surveys should be taken in more companies and also from other industries to ultimately validate the tool.

## 6. Conclusions

The assessment of the ability of enterprises to operate in individual phases of the global crisis allows for the development of risk tools and scales of organizational resilience. The paper develops a residual risk assessment tool based on the use of risk control measures related to Covid-19. However, the structure of the tool is universal and thus, it can be applicable to risk assessment of other threats and their impact on the resilience potential of an organization. On the basis of the survey, four measures related to the use of control measures against threats after the outbreak of the Covid-19 pandemic have been proposed. These are personal protective equipment (PPE) controls, administrative controls, engineering controls, and substitution controls. Due to the development of vaccines with 60–90% effectiveness, and their introduction to use after conducting the study, their effect is not included in the survey. On the other hand, anticipating vaccinations, they were treated as a control measure to eliminate the risk and, in the calculations, hypothetical responses were simulated. This resulted in a significant improvement in reducing the residual risk to a widely acceptable level. Using the survey results, we estimated averages of the response results and on their basis, we estimated the residual risks for individual types of enterprises according to the type of business and its size. Based on the calculations, a strong correlation was found between the potential of organizational resilience and the individual use of control measures. Therefore, the main finding of the survey proves that effective risk management builds organizational resilience in enterprises. The practical implications of the study allow the management staff to find out what aspects related to the use of control measures need to be paid attention to in order to minimize residual risk. Due to some limitations of the tool, additional surveys should be taken. Finally, this paper also provides knowledge about the benefits that companies can obtain by adopting the tool to combat COVID-19, mainly boosting the organizational resilience and minimizing the pandemic residual risk.

## Figures and Tables

**Table 1:** The probability scale. Source: Own study.

Scale	Probability	Description
1	Very rare	It may occur once a year
2	Rare	It may occur once every 6 months
3	Moderate	It may occur once every 3 months
4	Frequent	It may occur once a month
5	Very often	It may occur once a week

**Table 2:** The consequences scale. Source: Own study.

Category	H&L ^1^	F	E&S
1. Very small	There are no infected people. No individual has been quarantined.	Financial losses ˂ 1000 EURO.	An undetectable effect on the environment
2. Little	A small number of infected people (up to 10) but not seriously ill. A small number of people have been quarantined	Financial losses between 1000–10,000 EURO.	Low environmental impact with short-term effect.
3. Medium	Some require hospitalization (seriously ill) but no fatalities. Some people have been quarantined.	10,000 < Financial losses < 500,000 EURO. Difficulties in business continuity.	Some environmental effects, but short-term or small-term effects.
4. Big	A lot of hospitalized (seriously ill) people, a large number of people quarantined. Fatalities.	500,000 EURO ≤ Financial losses < 1,000,000 EURO. Special resources are needed to maintain business continuity	Long-term effects in the environment
5. Very big	A large number of seriously ill people. A large number of hospitalized patients. General and long-term quarantine. A large number of fatalities.	Financial losses ≥ 1,000,000 EURO. Breaks in business continuity.	High environmental impact and/or permanent damage

^1^ Description H&L—health and life F—finances in EURO E&S—environment and society.

**Table 3:** Scale of the hierarchy of risk control measures.

Scale	Control Measures	Description
1	Insufficient	PPE
2	Sufficient	Organizational
3	Good	Technical
4	Very good	Replacement
5	Perfect	Elimination

**Table 4:** Features of the research subjects.

Size of Company	Industrial	Other Sectors	All
All	33	33	66
Micro and small	3	13	16
Medium	13	13	26
Big	17	7	24

**Table 5:** Results of the internal consistency analysis.

No.	Rated Item	Mean	SD	Item-Total Correlations
1.	I have been provided with sufficient personal protective equipment by the company.	5.00	1.79	0.68
2.	Employees used masks or helmets at work to protect themselves from Covid-19 infection.	4.64	2.20	0.75
3.	At the very beginning of the internship, I was thoroughly trained in the field of health and safety requirements in the company.	5.68	1.42	0.62
4.	At the very beginning of the internship, I was informed in detail about the company’s rules related to Covid-19.	5.61	1.58	0.73
5.	The company scrupulously followed the procedures related to reporting cases of justified suspicions of Covid-19 disease.	5.15	1.50	0.70
6.	The company limited the number of employees staying in the workplace at the same time (shift system, rotation system, flexible working hours in order to avoid a large number of infected persons and maintaining business continuity in the event of an infection).	4.80	1.79	0.63
7.	Educational posters informing about the proper way of washing hands and other hygienic practices were placed in prominent places in the company.	5.62	1.60	0.67
8.	The company had rules of keeping social distance (information boards, increasing the distance of workplaces, limiting the presence of a certain number of people to the area of rooms, marking seats) “,	5.15	1.76	0.76
9.	Daily routine disinfection of frequently touched surfaces in the workplace (door handles, work tops, desks, keyboards, sinks, toilets, soap dispensers and others) was carried out on a regular basis.	5.23	1.72	0.67
10.	Near the entrance to the company and in many visible places on its premises, dispensers of skin disinfectants for employees are available for employees.	5.91	1.31	0.56
11.	The company created a competent crisis response team that coordinated the organization’s activities in the field of Covid-19.	4.68	1.79	0.75
12.	The management personnel effectively enforced the implemented regimes and procedures	5.11	1.55	0.78
13.	The company promoted remote work among employees wherever possible.	4.03	1.73	0.51
14.	The internship tutor was with me most of the time, and if he was unavailable at the moment, there was always someone to replace him	5.80	1.24	0.39
15.	The company has adapted to the pandemic situation and was able to seize the opportunities associated with it	4.97	1.41	0.21
16.	I did not have any obstacles in reporting the observed comments to the internship supervisor and entering them into the internship report.	5.53	1.07	0.28

**Table 6:** Scales of controls and the organizational resilience potential mean.

Scale	Item’s List	Industry	Other	1–49	50–250	250+	Total	α CR	It-Tot Cor.
PPE controls	1, 2	4.48	5.15	4.56	4.90	4.90	4.82	0.75	0.62
Administrative controls	3, 4, 7	5.54	5.74	5.04	5.64	6.03	5.64	0.77	0.58–0.63
Engineering controls	8, 9, 10	5.34	5.52	4.75	5.46	5.85	5.43	0.74	0.52–0.66
Substitution controls	6, 13	4.05	4.79	4.63	4.52	4.17	4.42	0.67	0.50
Elimination controls *	-	1.0	1.0	1.0	1.0	1.0	1.0	-	-
Total (calc.)	1, 2, 3, 4, 6, 7, 8, 9, 10, 13	2.50	2.71	2.48	2.63	2.66	2.61	0.90	0.89
Organizational resilience potential	5, 11, 12, 14, 15, 16	5.07	5.35	4.92	5.21	5.40	5.21	0.76	0.17–0.57

* Responses were assumed at level 1 on the Likert scale due to the inability to eliminate the risk during the study–time before vaccination started.

**Table 7:** Residual risk calculation, according to formula (1), for the assumed inherent risk index = 20.

Industrial	Others	1–49	50–250	250+	Total
1.97	1.76	1.99	1.84	1.81	1.86

**Table 8:** The results of the Spearman correlation between the total use of control measures and organizational resilience.

Variable	PPE Controls	Administrative Controls	Engineering Controls	Substitution Controls	Resilience	Total Controls
PPE controls	1.00	-	-	-	-	-
Administrative controls	0.61	1.00	-	-	-	-
Engineering controls	0.66	0.68	1.00	-	-	-
Substitution controls	0.59	0.43	0.58	1.00	-	-
Resilience	0.65	0.75	0.70	0.52	1.00	-
Total controls	0.87	0.75	0.85	0.82	0.75	1.00

## Data Availability

The study did not report any data publicly archived, datasets analyzed or generated during the study.
